# The effect of a multicomponent intervention on occupational fall‐related factors in older workers: A pilot randomized controlled trial

**DOI:** 10.1002/1348-9585.12374

**Published:** 2022-12-02

**Authors:** Yosuke Osuka, Yu Nofuji, Satoshi Seino, Kazushi Maruo, Hiroyuki Oka, Shoji Shinkai, Yoshinori Fujiwara, Hiroyuki Sasai

**Affiliations:** ^1^ Research Team for Promoting Independence and Mental Health Tokyo Metropolitan Institute of Gerontology Itabashi‐ku Japan; ^2^ Research Team for Social Participation and Community Health Tokyo Metropolitan Institute of Gerontology Itabashi‐ku Japan; ^3^ Department of Biostatistics, Faculty of Medicine University of Tsukuba Tsukuba Japan; ^4^ Department of Medical Research and Management for Musculoskeletal Pain, 22nd Century Medical and Research Center, Faculty of Medicine University of Tokyo Bunkyo‐ku Japan; ^5^ Graduate School of Nutrition and Health Science Kagawa Nutrition University Sakado Japan

**Keywords:** adherence, feasibility study, occupational safety, randomized controlled trial, safety

## Abstract

**Objectives:**

Multicomponent interventions reduce falls among community‐dwelling older adults. However, whether this strategy helps reduce occupational falls among older workers is unclear. This pilot trial tested the safety, adherence, and potential effectiveness of a multicomponent intervention for older workers.

**Methods:**

An assessor‐blind, parallel‐designed randomized controlled trial was conducted in five public employment agencies for seniors in Saitama, Japan. In total, 69 older adults who worked ≥4 days/month were randomly assigned to the intervention (*n* = 35) or control (*n* = 34) groups. The intervention group was provided a multicomponent intervention consisting of exercise, nutrition, and psychosocial programs once a week for 8 weeks. Safety was evaluated for all adverse events reported by participants. Adherence was assessed by rates for withdrawal/dropout, exercise practice, and nutritional diary completion. The primary outcome was a change in functional strength related to occupational falls. Secondary outcomes included changes in agility, balance, executive function, visuospatial ability, exercise self‐efficiency, dietary variety, social network, and functional capacity.

**Results:**

No adverse events were reported by participants. The median withdrawal/dropout, exercise practice, and nutritional diary completion rates were 0%, 80.4%–93.7%, and 100%, respectively. In the adjusted general linear model, the intervention group showed a non‐significant but clinically important improvement in functional strength (*P* value: .081, Cohen's *d*: 0.57) and significant improvements in agility, balance, and dietary variety compared to the control group.

**Conclusions:**

A multicomponent intervention for older workers would be a safe, acceptable, and effective strategy for improving risk factors for occupational falls.

## 1 INTRODUCTION

Policies promoting the employment of older adults are necessary to build a sustainable public social insurance system.^1^ However, we should recognize its negative side: the concern of increased occupational accidents involving older workers. For example, in major developed countries, including the U.S. and Japan, the incidence of occupational accidents among a whole generation is decreasing, while that in older generations is increasing.^2^, ^3^ In particular, occupational falls among older workers comprise a large proportion of all occupational accidents.^2^, ^3^ To extend the period when older workers can work safely (i.e., the “healthy working life expectancy”^4^), healthcare providers in occupational settings need to provide effective interventions to prevent occupational falls in older workers.^5^

Cumulative findings in geriatrics have provided solid evidence indicating that multicomponent interventions for community‐dwelling older adults help reduce falls during daily activities.^6^ However, whether such interventions would be an acceptable and helpful strategy for preventing occupational falls in older workers remains unclear. Our recent retrospective study found that modifiable intrinsic factors, such as reduced functional strength, poor balance, and visuospatial ability, were significantly associated with more occupational falls.^7^ Thus, a multicomponent intervention to modify such factors can help reduce occupational falls among older workers.

Whether a multicomponent intervention focusing on modifiable intrinsic factors reduces “incidence of occupational injuries or death” and “retirement due to fear of occupational accident” is a clinically important question. However, large‐scale clinical trials should be employed to answer this question. Pilot trials are an essential phase in evaluating the feasibility of future large‐scale clinical trials.^8^

This pilot trial reports on the safety, adherence, and potential effectiveness in reducing the occupational fall‐related factors of a multicomponent intervention for older workers. These preliminary data could provide reliable experience and helpful knowledge for planning a feasible large‐scale clinical trial in the future.

## 2 METHODS

### 2.1 Study design, setting, registration, and ethics

This study applied an assessor‐blind, randomized controlled, parallel‐group design. The settings were five Silver Human Resource Centers (SHRCs) in Saitama Prefecture. SHRCs are public employment agencies established under Japanese law, contributing to the well‐being of older adults and the community through volunteer, part‐time, and temporary jobs. There are 1335 SHRCs throughout Japan, with approximately 700 000 older adults registered.

The study protocol was developed following the guidelines of the CONSORT 2010 statement: extension to randomized pilot and feasibility trials.^9^, ^10^ We disclosed the protocol to University Hospital Medical Information Network Clinical Trials Registry before the study began (registration number: UMIN000044706, published date: 2021/06/30). This study started after review and approval by the Research Ethics Review Committee of the Tokyo Metropolitan Institute of Gerontology. Before beginning the baseline assessment, the study procedures were explained to all participants who provided their written consent. The study was conducted in accordance with the principles of the Declaration of Helsinki.

### 2.2 Participants

Participants were recruited through posters and flyers. The inclusion criteria were as follows: (1) age ≥60 years and registered with the SHRC; (2) worked ≥4 days/month; and (3) provided consent to participate in the study. The number of working days in the second criterion (≥4 days/month) was empirically determined based on the overall eligibility and feasibility of the study.

The exclusion criteria were as follows: (1) prohibited from exercising by their family physician; (2) had experienced angina, myocardial infarction, or cardiac surgery within the past 3 months; (3) had participated in the same assessment or intervention in the past; or (4) were judged as ineligible by the principal investigator. The fourth exclusion criterion allowed the principal investigator to decide when scientific validity and safety information were irregular or ambiguous or when the eligibility of a given candidate was difficult to determine (e.g., participants who had a strong surgical or internal complaint during exercise without experiencing angina, myocardial infarction, or cardiac surgery within the past 3 months).

### 2.3 Randomization and blinding

Participants were randomly allocated into the intervention and control groups at a 1:1 ratio. The block method was applied to the sequence of creating the allocation table (block size: 2), with sex and facilities as stratification factors. The principal investigator sent the participants' identification codes to a clinical statistician who was not involved in the intervention. The clinical statistician merged the pre‐generated allocation information with the participants' identification code. Assessors were blinded to the allocation of information during the assessment of physical and cognitive performance to minimize measurement bias.

### 2.4 Intervention

The intervention group was provided a class‐styled multicomponent intervention consisting of eight sessions of exercise, nutrition, and psychosocial programs once a week (Table 1). This was accomplished by modifying our earlier intervention program.^11^ We previously reported that the multicomponent intervention reduced frailty and improved functional capacity in community‐dwelling older adults.^11^ All programs were provided by pre‐trained and certified SHRC members. SHRC members attended 3 days of seminars to learn how to run the program and how to teach it effectively and safely. Participants in both groups were instructed not to change other aspects of their lifestyle during the intervention period. The control group was not provided any programs during the intervention period but was provided a similar intervention promptly after the follow‐up assessment, considering ethical concerns.

**TABLE 1:** Composition of a multifactorial intervention

Class	Exercise program	Nutrition program	Psychosocial program
1	Warm‐up, coordination exercise, stretching, and strength exercise. Repetition: 5	The importance of the intake of the 10 food groups	
2	Warm‐up, coordination exercise, stretching, and strength exercise. Repetition: 5		Tips for preventing falls at home
3	Warm‐up, coordination exercise, stretching, and strength exercise. Repetition: 7	The roles and practical information of protein	
4	Warm‐up, coordination exercise, stretching, and strength exercise. Repetition: 7	The roles and practical information of calcium	Game‐style listening skills
5	Warm‐up, coordination exercise, stretching, and strength exercise. Repetition: 7	The roles and practical information of fat	
6	Warm‐up, coordination exercise, stretching, and strength exercise. Repetition: 10	The roles and practical information of vitamins	
7	Warm‐up, coordination exercise, stretching, and strength exercise. Repetition: 10	The roles and practical information of carbohydrate	Rediscovering the charm of the community
8	Warm‐up, coordination exercise, stretching, and strength exercise. Repetition: 10	Find foods you are lacking in your daily diet	

#### 2.4.1 Exercise program

The exercise program was provided in the following order: (1) warm‐up (5 min), (2) coordination exercise (10 min), (3) stretching (5 min), and (4) strength exercise (20 min). The warm‐up consisted of stepping, arm swings, moving shoulders up and down, and shoulder rotations. The coordination exercise is provided to enhance the interaction of motor and cognitive tasks that occur during work (e.g., exercises in which movements are performed as quickly as possible that are different from those indicated by visual information and exercises in which hands and feet are moved in different rhythms). Stretching included exercises such as standing on tiptoe and chest stretching to improve the flexibility of muscles, tendons, and joints. Finally, strength exercises included calf raises, toe raises, and knee extensions to improve work activities. Participants were asked to perform these exercises until they felt “somewhat hard.”

Participants were also asked to perform seven exercises (toe raises, calf raises, knee ups, knee extensions, squats, hip twists, and oral exercises) with 10–20 repetitions/day at their homes and walk for 20 min/day as aerobic exercise and record that information in exercise diaries. If they felt unwell or felt that the training intensity was too high, they were instructed to stop exercising or reduce the number of repetitions.

#### 2.4.2 Nutrition program

Certified SHRC members explained in a picture‐story style, the importance of eating the 10 food groups (such as fats and oils, meat, and green and yellow vegetables) and the roles and practical information on nutrients such as protein and calcium. The participants were then asked to record their intake score for the 10 food groups at home daily.

#### 2.4.3 Psychosocial program

The psychosocial program consisted of “tips for preventing falls at home,” “game‐style listening skills,” and “rediscovering the charm of the community.” Certified SHRC members explained these to the participants using posters. Tips for preventing falls at home were provided as a quiz to help participants learn about fall prevention, such as bones that can be easily broken by falls or locations where falls are likely to occur. Self‐introduction was practiced in the form of games to communicate well with others. In the “rediscovering the charm of the community” session, the traditional Japanese card game “karuta” was used to explain the characteristics of the community.

#### 2.4.4 Adherence

Adherence was evaluated based on the “withdrawal/dropout rates,” “eight exercise practice rates at home,” and “completion rate of the nutrition diary.” The withdrawal/dropout was defined as those who withdrew because of unavoidable circumstances (e.g., hospitalization or moving) or dropped out during the intervention period. The exercise participation rate was calculated by summing the number of days each exercise program was implemented (the number of days when a daily record confirmed implementation at least once) and dividing by the number of intervention days. Finally, the completion rate of the nutrition diary was determined by summing the number of days recorded and dividing them by the number of intervention days.

#### 2.4.5 Adverse events

All adverse events, including illness, disability, death, infection, unintended signs, clinically significant changes in laboratory values, symptoms, or worsening complications, were evaluated regardless of the causal relationship with the intervention. The staff of the SHRC recorded the date and nature of the adverse event when the participants reported them.

### 2.5 Effectiveness assessment

The primary outcome was a change in functional strength, and the secondary outcomes were a change in agility, balance, executive function, visuospatial ability, exercise self‐efficiency, dietary variety, social network, and functional capacity. Reduced functional strength, poor standing balance, and poor visuospatial ability were associated with a significantly higher probability of occupational fall incidence, indicating that these assessments may be helpful as surrogate markers for occupational falls.^7^ We had planned to assess depressive symptoms with the Geriatrics Depression Scale, but this assessment was not performed because the COVID‐19 pandemic necessitated a reduction in survey time. Motor and cognitive assessments were performed by certified SHRC members who were not involved in the intervention.

#### 2.5.1 Functional strength

Functional strength was assessed by the five‐repetition sit‐to‐stand test (5SST).^12^ The participants were instructed to stand up and sit down from a chair (42.5 × 51.0 × 51.0 cm) five times as quickly as possible, with arms crossed in front of the chest and legs shoulder‐width apart. The assessor measured the time required from the start cue to the fifth sitting.

#### 2.5.2 Agility

Agility was assessed using the alternate step test (AST).^12^ The participants were instructed to place one foot on a stepladder (20 cm high) eight times as quickly as possible, alternating the left and right foot. The assessors measured the time required from the start cue to the eighth time the foot touched the ground.

#### 2.5.3 Balance

Balance was assessed using the near tandem balance test (NTBT).^12^ Participants were instructed to stand on a piece of laminated paper with a near‐tandem footprint (one foot in front of the other with the heel of the front foot and toes of the back foot 2.5 cm apart) printed on it. The assessors measured the time from when the participants closed their eyes until they lost balance or reached 30 s. If the first measurement was <5 s, a second measurement was allowed, and the maximum value was used for analysis.

#### 2.5.4 Executive function

Executive function was assessed using a brief version of Trail Making Test‐B (TMT‐B).^13^ Participants were instructed to connect circles with numbers (1–5) and letters (a–o) in alternating and ascending order (i.e., 1‐a‐2‐i…). The assessors judged the participants as “correct” if they could connect the lines as instructed and “incorrect” if they could not.

#### 2.5.5 Visuospatial ability

Visuospatial ability was assessed using a 3D cube copy test.^13^ Participants were instructed to copy the cube as accurately as possible. The assessors judged the participants as “correct” if they could copy the cube accurately and “incorrect” if they could not.

#### 2.5.6 Exercise self‐efficacy

The Home‐Exercise Barrier Self‐Efficacy Scale was used to assess confidence in regularly practicing the exercise at home.^14^ Participants were asked to respond to six questions (e.g., “I am confident that I exercise even when I am tired” or “I am confident that I exercise even when I don't have time”) using a 5‐point scale from “1: not confident at all” to “5: definitely confident.” The scores range from 6 to 30, with higher scores indicating a greater confidence in practicing the exercise at home.

#### 2.5.7 Dietary variety

Participants were asked to answer the frequency of consumption of 10 food groups (seafood, meat, eggs, milk, soy products, green and yellow vegetables, seaweed, potatoes, fruits, and oils and fats) using a 4‐point scale: “almost every day,” “once every two days,” “once or twice a week,” or “rarely.” Next, dietary variety scores (DVS) were calculated by summing the scores of “almost every day” as 1 point and other responses as 0 points.^15^ DVS range from 0 to 10, with higher scores indicating a greater variety of dairy foods.

#### 2.5.8 Social network

Social networks were assessed using the Japanese version of the abbreviated Lubben Social Network Scale (LSNS‐6).^16^ The LSNS‐6 comprises six questions. First, participants were asked about the number of family members and friends who provide emotional support in their daily lives using a 6‐point scale ranging from “none: 0 points” to “five: 5 points.” The LSNS‐6 score range is 0–30, with higher scores indicating a lower likelihood of social isolation and stronger social connections.

#### 2.5.9 Functional capacity

Functional capacity was assessed using the Kaigo‐Yobo Check‐List.^17^ Participants were asked to answer 15 questions regarding items such as confinement, falls, and malnutrition, using a 2‐point scale: “yes; 1 point” or “no; 0 points.” The scores range from 0 to 15 points, with higher scores indicating declined functional capacity.

### 2.6 Sample size

The multicomponent intervention was estimated to provide a small‐to‐moderate effect size (Cohen's *d* = 0.2 to 0.6) in the definitive (phase III) study. A previous study reported that the sample size in a pilot study required to examine these effect sizes in a phase II study is ≥55.^18^ We expected a dropout rate of approximately 10% (*n* = 8) after allocation and 20% (*n* = 20) of absences in the baseline assessment after recruitment and thus recruited up to 100 participants.

### 2.7 Statistical analysis

Adherence and adverse events are shown using descriptive statistics. To identify the potential effectiveness of the multicomponent intervention, we compared the change in outcome values (the difference between baseline and follow‐up measurements) between the intervention and control groups using a general linear model with baseline values, sex, and facility units as covariates. The estimated values are shown as adjusted mean difference (95% confidence interval: CI). The TMT‐B and 3D cube copy tests were compared for the percentage of correct answers for each group in the follow‐up assessment. Missing data were processed using a listwise method. Effectiveness outcomes were analyzed based on the intention‐to‐treat principle, i.e., participants who (1) met all eligibility criteria and (2) had completed both baseline and follow‐up assessments were included in the analysis.^19^ All analyses were performed using R version 4.1.2 (R Foundation, Vienna, Austria). The statistical significance level was set at 5%.

## 3 RESULTS

### 3.1 Enrollment

**FIGURE 1:**
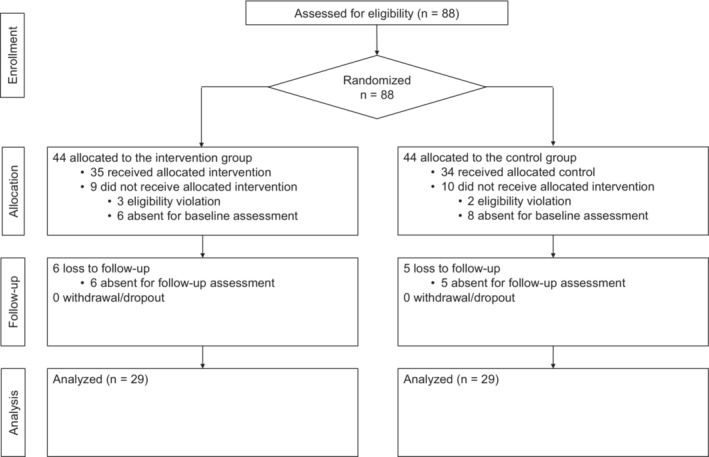
Flow diagram of the study participants.

Figure 1 shows the flow from recruitment to analysis. After the recruitment period, 88 individuals who met the eligibility criteria were invited to participate in this trial. A total of 69 participants were included in the study. However, 5 participants (3 and 2 in the intervention and control groups, respectively) and 14 participants (6 and 8 in the intervention and control groups, respectively) were excluded because of eligibility violations and absence at the baseline survey. After excluding 11 (6 and 5 in the intervention and control groups, respectively) who were absent from follow‐up, 58 (29 and 29 in the intervention and control groups, respectively) were analyzed. Owing to the widespread prevalence of COVID‐19, two sessions were canceled at one SHRC.

### 3.2 Baseline characteristics

Table 2 summarizes the baseline characteristics of all participants in the intervention and control groups. The median [interquartile range: IQR] age of all participants was 73 [71–77] years. The proportion of women was 50.7% (*n* = 35). The median [IQR] number of days worked per month and median [IQR] hours worked per day for the participants were 10 days [7.5–13 days] and 4 h [2.5–5 h], respectively.

**TABLE 2:** Baseline characteristics of study participants

	All	Intervention group	Control group
	*n* = 69	*n* = 35	*n* = 34
Age, years	73 [71, 77]	73 [70, 77]	74 [72, 79]
Female	35 (50.7)	19 (54.3)	16 (47.1)
Working days, day/month	10 [7.5, 13]	10 [7, 13]	10 [8, 12]
Working hours, hours/day	4 [2.5, 5]	4 [3, 5]	3.3 [2, 5]
Hypertension	27 (39.1)	12 (34.3)	15 (44.1)
Diabetes	8 (11.6)	5 (14.3)	3 (8.8)
Heart disease	7 (10.1)	3 (8.6)	4 (11.8)
Stroke	1 (1.4)	1 (2.9)	0 (0.0)
Eye disease	15 (21.7)	7 (20.0)	8 (23.5)
Knee arthritis	3 (4.3)	2 (5.7)	1 (2.9)

*Note*: Data are presented as median [interquartile range] or *n* (%).

### 3.3 Adherence and safety

There were no withdrawals/dropouts in either group. Table 3 shows adherence to the exercise and nutrition program. Exercise and nutrition diaries were collected from 32 of the 35 participants. The median practice rate of the eight home exercise programs (e.g., toe rises) was 80.4%–93.7% and the median completion rate [IQR] of the nutrition diary was 100% [87.5%–100%]. The median participation rate [IQR] of the classes was 87.5% [75%–100%]. Participants reported no adverse events during the intervention period.

**TABLE 3:** Adherence to the exercise and nutrition program, *n* = 32

Exercise diary	
Toe raises, %	93.7 [85.7–100]
Calf raises, %	85.7 [93.7–99.2]
Knee ups, %	91.1 [82.2–99.1]
Knee extensions, %	91.1 [82.2–98.3]
Squats, %	90.5 [74.1–98.2]
Hip twists, %	87.5 [77.7–95.5]
Oral exercises, %	87.5 [64.3–100]
Walking, %	80.4 [44.5–92.5]
Nutrition diary	100 [87.5–100]

*Note*: Data are shown as the median [interquartile range]. The practice rate for each exercise program was determined by summing the days on which the program was practiced at least once divided by the intervention days. The completion rate of the nutrition program was obtained by summing the days of record divided by the intervention days. Three participants did not provide exercise and nutrition diaries.

### 3.4 Potential effectiveness

Table 4 presents the results of the effectiveness outcomes. A non‐significant difference between the groups [95% CI] was noted in the 5SST (primary outcome) results (−1.0, [−2.1, 0.1]; *P* value: .081; Cohen's *d*: 0.57), which favored the intervention group. There were significant differences in agility, balance, and dietary variety between the groups; the intervention group consumed less time for AST and more time for NTBT and had higher DVS than the control group (*P* values <.05, Cohen's *d*: 0.24–0.75). No significant differences were found between the intervention and control groups in the percentage of correct answers on the TMT‐B and 3D cubic test at the follow‐up assessment (TMT‐B: intervention group, 82.8% vs. control group 93.1%, *P* = .423; 3D cubic test: intervention group 86.2% vs. control group 82.8%, *P* = 1.000).

**TABLE 4:** The potential effectiveness of a multicomponent intervention

	Intervention group, *n* = 29			Control group, *n* = 29			Difference^a^	*P* values	Cohen's *d*
	Baseline	Follow‐up	Change	Baseline	Follow‐up	Change			
5SST, sec	10.4 (5.1)	8.1 (2.6)	−2.3 (4.0)	9.1 (1.9)	8.6 (2.3)	−0.5 (1.9)	−1.0 (−2.1, 0.1)	.081	0.57
AST, sec	7.8 (2.1)	6.9 (1.2)	−0.8 (1.3)	7.5 (1.4)	7.5 (1.6)	−0.1 (1.1)	−0.6 (−1.1, −0.1)	.017	0.58
NTB, sec	27.1 (7.0)	29.8 (1.9)	2.7 (5.9)	25.0 (9.1)	26.3 (8.2)	1.3 (5.7)	2.4 (0.1, 4.6)	.046	0.24
ESE, score	20.4 (3.6)	20.7 (3.9)	0.3 (3.6)	20.0 (3.7)	20.3 (4.1)	0.0 (4.2)	0.7 (−1.2, 2.7)	.468	0.08
LSNS, score	18.0 (5.6)	17.2 (4.6)	−0.7 (4.8)	16.6 (5.7)	16.8 (5.9)	0.2 (5.0)	−0.5 (−2.7, 1.8)	.686	0.18
KYCL, score	1.2 (1.5)	1.0 (1.6)	−0.2 (1.3)	1.0 (1.3)	1.0 (1.2)	0.0 (1.1)	−0.1 (−0.6, 0.5)	.853	0.17
DVS, score	4.2 (2.2)	5.5 (2.7)	1.3 (2.0)	3.8 (2.1)	3.7 (2.5)	−0.1 (1.7)	1.6 (0.6, 2.5)	.002	0.75

*Note*: Baseline and follow‐up assessment values and change in values are shown as means (standard deviations) and adjusted between‐group differences in change values are shown from means (95% confidence intervals).

Abbreviations: 5SST, five‐repetition sit‐to‐stand test; AST, alternate step test; DVS, dietary variety scores; ESE, exercise self‐efficacy; KYCL, Kaigo‐Yobo Check‐List; LSNS, Lubben Social Network Scale; NTBT, near tandem balance test.

^a^ Adjusted for baseline values, sex, and facility units.

## 4 DISCUSSION

To our knowledge, no randomized controlled trials have evaluated the effects of intervention strategies focused on reducing occupational falls in older workers. This pilot study is the first to report the safety, adherence, and potential effectiveness of a multicomponent intervention for older workers. As a result, no adverse events were reported by participants. The median rates for withdrawal/dropout, the eight exercise practices, and completion of the nutrition diary were 0%, 80.4%–93.7%, and 100%, respectively. The intervention group showed a non‐significant but clinically important improvement in functional strength compared to the control group. Additionally, the intervention group showed significant improvements in balance, agility, and dietary variety compared to the control group. These preliminary results indicate that our multicomponent intervention is a safe, acceptable, and effective strategy for improving occupational fall risk factors among older workers.

Our results showed that a moderate‐intensity, controlled exercise program for older workers is safe. Notably, although exercise programs reduce the risk of falls in older adults, they are associated with a risk of falls and musculoskeletal complaints during or after the training.^20^ For example, a clinical trial that provided home‐based high‐intensity resistance training to frail older adults reported that the risks from exercise‐induced musculoskeletal injury outweighed the benefits of the intervention.^21^ In addition, a recent large clinical trial reported that the multicomponent intervention group fell more than the control group among participants with severe frailty.^22^ Thus, whether multicomponent interventions are safe for physically frail older workers needs to be tested in future clinical trials.

The withdrawal/dropout rate in our previous 12‐week multicomponent intervention was 10.5% (no withdrawal/dropout was seen in this study).^11^ A study examining definitions of adherence to various exercise classes suggests that adherence is often defined as high when the participation rate in the class is ≥75% (the rate in our study was 87.5).^23^ Sjösten et al. reported that older adults with higher physical, cognitive, and psychological function have better adherence to multicomponent interventions.^24^ The reason the adherence to our intervention was better than that of previous studies may be because the participants had higher functional capacity than the general older population. Moreover, because co‐workers delivered this intervention in the workplace, it may have increased adherence through a social network that reinforced behavioral change.^25^, ^26^ However, our interventions were shorter and less frequent than in previous studies. Therefore, future clinical studies should clarify whether high adherence can be maintained when the duration of the intervention is extended, or the frequency of the intervention is increased.

The study showed clinically important improvement in known fall risk factors, including functional strength, balance, and agility (Cohen's *d*: 0.24–0.58).^27^ Our results support previous findings reporting that a multicomponent intervention helps improve functional health and lifestyle in community‐dwelling older adults.^11^ Our prior data show that a 1‐s fast 5SST results in approximately a 10% reduction in the rate of occupational falls.^7^ The average net benefit of the multicomponent intervention for the five‐chair sitting test was 1.0 s (Table 4). Thus, large, long‐term clinical trials designed to detect a 10% reduction in occupational fall rate could confirm the clinical utility of multicomponent interventions in the future.

Trained and certified SHRC members provided multicomponent interventions and assessments. Implementing effective interventions and assessments by non‐specialists has critical clinical implications because of limited specialist resources in the public health care system.^28^ A mutually supportive framework may be a sustainable occupational fall prevention strategy. The multicomponent intervention was provided based on our findings in frailty prevention^11^; however, whether this is a better approach for occupational fall prevention is unknown. For example, while exercise intervention, especially in balance plus functional exercise, has the strongest evidence to support its use as a preventive strategy for falls in community‐dwelling older adults, the effectiveness of nutritional interventions, such as vitamin D supplementation, is not clear.^29^ Our current understanding suggests that limiting the occupational falls preventive program to exercise intervention alone may enhance the cost‐effectiveness and scalability of the main trial.

This study has some limitations. Owing to the small sample size, this pilot trial could not assess whether the safety, adherence, and efficacy of the intervention differed by participant characteristics (e.g., sex, age, or job description). Thus, future, large‐scale, long‐term clinical trials should apply subgroup analyses to assess heterogeneity for these outcomes. It is also unclear whether this strategy can be generalized to older workers with full‐time jobs or other regions or countries since all participants worked temporary or part‐time jobs and were recruited from a specific area of Japan. Therefore, future studies should test the generalizability of the results of this study to different populations.

## 5 CONCLUSION

This pilot trial showed that a multicomponent intervention comprising exercise, nutrition, and psychosocial programs is a safe, acceptable, and helpful strategy for older workers to improve occupational fall factors. However, whether this strategy has clinically significant implications (e.g., reduction in occupational falls with injury in older workers) should be tested in large‐scale, long‐term clinical trials.

## AUTHOR CONTRIBUTIONS

YO, YN, SSeino, KM, HK, SShinkai, YF, and HS conceived the ideas; YO collected the data; KM planned the analysis; and HS analyzed the data. All authors prepared the manuscript, interpreted the results, and confirmed the final version of the manuscript.

## DISCLOSURES

*Approval of the Research Protocol*: The study protocol was disclosed to University Hospital Medical Information Network Clinical Trials Registry before the study began (registration number: UMIN000044706, published date: 2021/06/30). This study started after review and approval by the Research Ethics Review Committee of the Tokyo Metropolitan Institute of Gerontology. *Informed Consent*: All participants provided written consent. *Registry and Registration No*: University Hospital Medical Information Network Clinical Trials Registry (registration number: UMIN000044706, published date: 2021/06/30). *Animal Studies*: N/A. *Conflict of Interest*: The SHRCs provided lecture fees to YO, YN, and SS. The other authors declare no conflict of interests for this article.

## Data Availability

Research data are not shared.
